# Long-term effects of adjuvant treatment for breast cancer on carotid plaques and brain perfusion

**DOI:** 10.1007/s10549-020-05990-y

**Published:** 2020-11-05

**Authors:** Vincent Koppelmans, Kimberly D. van der Willik, Berthe M. P. Aleman, Flora E. van Leeuwen, Maryam Kavousi, Banafsheh Arshi, Meike W. Vernooij, M. Arfan Ikram, Sanne B. Schagen

**Affiliations:** 1 Department of Epidemiology, Erasmus MC University Medical Center, Rotterdam, The Netherlands; 2 Department of Psychosocial Research & Epidemiology, Netherlands Cancer Institute/Antoni Van Leeuwenhoek Hospital, Plesmanlaan 121, 1066 CX Amsterdam, The Netherlands; 3 Department of Psychiatry, University of Utah, Salt Lake City, USA; 4 Department of Radiation Oncology, Netherlands Cancer Institute/Antoni Van Leeuwenhoek Hospital, Amsterdam, The Netherlands; 5 Department of Radiology and Nuclear Medicine, Erasmus MC University Medical Center, Rotterdam, the Netherlands; 6 Department of Psychology, Brain and Cognition, University of Amsterdam, Nieuwe Achtergracht 129-B, 1018 WS Amsterdam, The Netherlands

**Keywords:** Breast cancer, Radiotherapy, Chemotherapy, Brain perfusion, Carotid plaques, Intima-media thickness

## Abstract

**Purpose:**

Breast cancer treatment has been associated with vascular pathology. It is unclear if such treatment is also associated with long-term cerebrovascular changes. We studied the association between radiotherapy and chemotherapy with carotid pathology and brain perfusion in breast cancer survivors.

**Methods:**

We included 173 breast cancer survivors exposed to radiotherapy and chemotherapy, assessed ± 21.2 years after cancer diagnosis, and 346 age-matched cancer-free women (1:2) selected from the population-based Rotterdam Study. Outcome measures were carotid plaque score, intima-media thickness (IMT), total cerebral blood flow (tCBF), and brain perfusion. Additionally, we investigated the association between inclusion of the carotid artery in the radiation field (no/small/large part), tumor location, and these outcome measures within cancer survivors.

**Results:**

Cancer survivors had lower tCBF (− 19.6 ml/min, 95%CI − 37.3;− 1.9) and brain perfusion (− 2.5 ml/min per 100 ml, 95%CI − 4.3;− 0.7) than cancer-free women. No statistically significant group differences were observed regarding plaque score or IMT. Among cancer survivors, a large versus a small part of the carotid artery in the radiation field was associated with a higher IMT (0.05, 95%CI0.01;0.09). Also, survivors with a right-sided tumor had lower left carotid plaque score (− 0.31, 95%CI − 0.60;− 0.02) and higher brain perfusion (3.5 ml/min per 100 ml, 95%CI 0.7;6.2) than those with a left-sided tumor.

**Conclusions:**

On average two decades post-diagnosis, breast cancer survivors had lower tCBF and brain perfusion than cancer-free women. Also, survivors with a larger area of the carotid artery within the radiation field had a larger IMT. Future studies should confirm if these cerebrovascular changes underlie the frequently observed cognitive problems in cancer survivors.

## Introduction

Breast cancer patients are at an increased risk of developing cardiovascular diseases due to effects of cancer treatment on the vascular system [1–3]. Adjuvant chemotherapy has been associated with vascular damage [4], specifically with narrowing of the vascular lumen as a result of thickening of the vessel wall through endothelial damage [5]. In addition, it has been related to cardiotoxicity through injury of cardiac myocytes and antimetabolites, which is associated with myocardial ischemia [6]. Adjuvant radiotherapy for breast cancer has also been linked to vascular pathology [7], including carotid stenosis [8, 9], and carotid stiffness [10], as well as to an increased risk of stroke [11]. In addition, breast cancer patients may have a higher risk of congestive heart failure and myocardial infarction that can persist up to 20 years after treatment [1, 12–14]. However, the synergistic effects of chemotherapy and radiotherapy on vascular pathology in breast cancer patients that may arise due to accumulation of vascular damage remain largely unknown.

Cardiovascular diseases including carotid pathology can result in changes in total cerebral blood flow (tCBF) [15]. In turn, a preclinical study has shown that disrupted tCBF in mice can lead to cognitive deficits [16] and lower tCBF has been associated with accelerated cognitive decline and dementia in human [17]. As yet, it is unknown if the potential cardiovascular side effects of breast cancer itself and breast cancer treatment are associated with disruptions in tCBF and therefore with brain perfusion. Such knowledge is of particular interest because it could contribute to the understanding of the well-documented brain structural alterations and cognitive deficits that are prevalent in about 20% to 30% of cancer survivors [18].

We have previously shown that such structural brain alterations including reductions in total brain volume and gray matter volume, and cognitive deficits can occur up to 20 years after cessation of cancer treatment in breast cancer survivors who were treated with radiotherapy and subsequent CMF (Cyclophosphamide-Methotrexate-Fluorouracil) chemotherapy [19, 20]. In the current study that uses the same study population, we characterized the combined effects of cancer itself, radiotherapy, and chemotherapy, on atherosclerotic carotid disease and tCBF by comparing these breast cancer survivors to a 1:2 age-matched population-based, cancer-free reference group. To gain further insight into the contribution of regional radiotherapy on carotid pathology, we assessed the association between carotid atherosclerosis and radiation fields. The prevalence of cardiovascular diseases may differ between patients with left- and right-sided breast tumors [1]. Also, due to left–right differences in anatomy, a larger part of the left carotid artery may lie in the radiation field than of the right artery. We therefore determined the association between tumor location (left or right side) and carotid plaque score.

## Materials and methods

### Participants

#### Breast cancer survivors

We identified women with a history of unilateral, invasive breast cancer from the registries of the Netherlands Cancer Institute/Antoni van Leeuwenhoek Hospital and the Daniel den Hoed Cancer Clinic of the Erasmus Medical Center. Women were selected for the current study if they had been treated with both post-surgical radiotherapy and six cycles of adjuvant CMF chemotherapy (Cyclophosphamide 100 mg/m^2^ on days 1–14; Methotrexate 40 mg/m^2^ on days 1 and 8; 5-Fluorouracil 600 mg/m^2^ on days 1 and 8) between 1976 and 1995. The radiotherapy regimen depended on type of surgery and disease stage and was classified into one or more of the following fields: axillary, breast, chest wall, internal mammary chain, McWhirter, or supraclavicular radiation.

Breast cancer survivors were eligible if they were between 50 and 80 years of age at time of selection, if invasive breast cancer was their first and only malignancy, if they had remained cancer-free since treatment for breast cancer, and if they had sufficient command of the Dutch language. Exclusion criteria were use of adjuvant endocrine therapy and magnetic resonance imaging (MRI) contraindications.

A detailed overview of the participant inclusion has been described previously [21]. In short, 195 (67%) of the 291 eligible breast cancer survivors agreed to participate and were assessed between October 2008 and October 2009. Of the 195 women who participated, in four of them, no tCBF data were available because they had not completed the MRI examination due to claustrophobia. In another four participants, the ultrasound images of the carotid arteries of either one or two vessel beds were unusable. Hence, total plaque score could not be calculated for these subjects. Lastly, intima-media thickness (IMT) was not measured for five participants. Finally, 182 participants were available for analyses.

#### Population-based reference women

Women without a history of cancer were selected from the prospective population-based Rotterdam Study [22, 23]. As of 2008, the study includes 14,926 participants. By the end of the inclusion period of chemotherapy-exposed breast cancer survivors (October 2009), 1337 female participants of the Rotterdam Study had undergone complete carotid artery ultrasound assessment and a brain MRI [23]. Each breast cancer survivor was randomly matched to two out of these 1,337 cancer-free women based on age at time of carotid artery ultrasound (age range ± 4 years). Nine out of 182 breast cancer survivors could not be matched. We chose to match two cancer-free women per breast cancer survivor to limit the number of unmatched participants, which might induce selection bias.

### Methods

All examinations for both the breast cancer survivors and the reference women took place at the research center of the Rotterdam Study and were conducted by the same technicians. Breast cancer survivors were assessed between October 2008 and October 2009, and reference women were examined between April 2006 and August 2009.

#### Carotid artery ultrasound

Ultrasonography of both carotid arteries was performed with a 7.5-MHz linear-array transducer and a duplex scanner (EnVisor; Philips Medical Systems Nederland B.V, Eindhoven, Netherlands). Plaques, defined as focal widenings of the vessel wall relative to adjacent segments with protrusion into the lumen composed of either only calcified deposits or a combination of calcified and noncalcified material, were examined at six sites for both the left and right side including the anterior (near) and posterior (far) walls of the (a) internal carotid artery, (b) carotid bifurcation, and (c) common carotid artery [24]. A weighted plaque score ranging from 0 to 6 was computed by adding the number of sites at which a plaque was detected, divided by the total number of sites for which an ultrasonographic image was available and multiplied by 6 (the maximum number of sites). IMT was measured on a longitudinal, 2-dimensional ultrasound image of the distal common carotid artery, on which the near and far walls were displayed as two bright white lines separated by a hypoechogenic space [25]. IMT was defined as the distance between the leading edge of the far wall – displayed as the first bright line – and the leading edge of the near wall, i.e., the second bright line. The mean IMT was calculated as the average of three measurements of both the left and right carotid arteries.

#### MRI acquisition and processing

MRI was performed on a 1.5-T MRI scanner (General Electric Healthcare, Milwaukee, Wisconsin). During the study period, no software or hardware upgrades were performed. Breast cancer survivors and reference women were scanned using the same MRI scanner.

Our full scan protocol has been described in detail previously [23]. In short, for tCBF measurement, 2D phase-contrast imaging was performed. First, a sagittal 2D phase-contrast MRI angiographic scout image was performed. On this scout image, a transverse imaging plane perpendicular to both the precavernous portion of the internal carotid arteries and the middle part of the basilar artery was chosen for an axial 2D phase-contrast image (repetition time = 20 ms, echo time = 4 ms, field of view = 19 cm^2^, matrix = 256 × 160, flip angle = 8°, number of excitations = 8, bandwidth = 22.73 kHz, velocity encoding = 120 cm/s, slice thickness = 5 mm).

As previously described, we calculated flow from the phase-contrast images using interactive data language-based custom software (Cinetool version 4, General Electric Healthcare, Milwaukee, WI, USA) [23]. Regions of interest (ROIs), encompassing the entire lumen of the vessel, were drawn manually around both carotids and the basilar artery at the level of the clinoid segment on the phase-contrast images. The mean signal intensity in each ROI reflects the flow velocity in the vessel (cm/seconds). Flow (in ml/s) was calculated by multiplying the average velocity with the cross-sectional area of the vessel. To calculate total cerebral blood flow (tCBF, in ml/min), flow rates for the carotid arteries and the basilar artery were summed and multiplied by 60 s/minute. Two independent, experienced technicians performed all manual ROI drawing and flow measurements (inter-rater correlations > 0.94 for all vessels).

Total CBF strongly depends on the amount of brain tissue [26]. To account for this, we calculated brain perfusion (in ml/min per 100 ml) by dividing tCBF (ml/min) by brain volume (ml) and multiplying the obtained result by 100. Brain volume was automatically obtained from three high-resolution axial MRI sequences that were acquired for each participant: a T1-weighted three-dimensional fast radio frequency spoiled gradient recalled acquisition in steady state with an inversion recovery prepulse (FASTSPGR-IR); a proton density-weighted sequence; and a fluid-attenuated inversion recovery sequence (FLAIR). Preprocessing steps and the classification algorithm that were used to extract total brain volume (TBV) from these three sequences have been described in detail elsewhere [27]. In short, voxels were segmented into either: gray matter, white matter, cerebrospinal fluid, or background. The number of gray matter and white matter voxels were summed up and multiplied by the volume per voxel in mm^3^ to obtain total brain volume.

#### Demographics

Information on potential confounders was collected for all participants. Body mass index (BMI) was calculated from height and weight measurements (kg/m^2^). Sitting diastolic and systolic blood pressures (mmHg, average of two assessments) were measured on the right arm with a random-zero sphygmomanometer [26]. Self-reported data on age at menopause, diabetes mellitus, smoking status (current, ever, never), and education level (primary education; lower education (lower/intermediate general education or lower vocational education); intermediate (intermediate vocational education or higher general education); and higher (higher vocational education or university) were obtained. In addition, information on use of antihypertensive medication, anticoagulant medication, and lipid-lowering medication was collected.

### Analyses

Analysis of variance (ANOVA, continuous variables) and *χ*^2^ tests (categorical variables) were used to compare characteristics of breast cancer survivors and population-based reference women.

We used negative binomial regression to compare the distribution of plaque scores between groups (count variable, range 0–12), and linear regression models to compare groups on IMT (continuous), tCBF (continuous), and brain perfusion (continuous). Even though we matched the breast cancer survivors and controls on age at carotid artery ultrasound, we corrected all analyses for age to account for potential residual confounding by age. In addition, all analyses were corrected for BMI. In an extended model, ‘Model II,’ we additionally corrected for prevalence of diabetes mellitus, smoking status, use of anticoagulant medication and lipid-lowering medication, and education level. We adjusted for education level because of the different distribution of education level between groups in the current study and its association with cardiovascular diseases in general [28]. Note that all potential confounders were measured at time of assessment of the outcomes and not at time of cancer diagnosis and treatment. We therefore considered age at menopause, systolic and diastolic blood pressure, and use of antihypertensive medication as potential mediators rather than potential confounders, and did therefore not correct for these factors [29, 30]. Lastly, for the analyses on tCBF and brain perfusion, we additionally corrected for total plaque score and total IMT in Model III to determine whether any association was explained by plaque score or IMT.

Within the group of breast cancer survivors, we investigated if the degree to which the carotid artery was included in the radiation field was associated with plaque scores (negative binomial regression analysis), IMT, tCBF, or brain perfusion (linear regression analysis). We therefore classified field of radiotherapy in (a) carotid artery was not the in radiation field (only axillary, breast, or chest wall radiation); (b) a small part of the carotid artery was in the radiation field (internal mammary chain radiation, with or without axillary, breast, or chest wall radiation); and (c) a large part of the carotid artery was in the radiation field (McWhirter or supraclavicular with or without internal mammary chain, axillary, breast, or chest wall radiation). For these analyses, we used the same models as used when comparing breast cancer survivors with cancer-free reference women. Individuals with a small part of the carotid artery in the radiation field were selected as the reference group because this was the most common type of treatment (see Table 1).

**Table 1:** Baseline characteristics of the study population

Characteristic	Breast cancer survivors (n = 173)	Cancer-free reference women (n = 346)	*P*
Age, years, mean (SD)	63.8 (6.5)	61.7 (6.2)	< .001
Body mass index, kg/m^2^, mean (SD)	26.7 (4.6)	27.4 (4.6)	.11
Systolic blood pressure, mmHg^a^, mean (SD)	139 (19)	131 (19)	< .001
Diastolic blood pressure, mmHg^a^, mean (SD)	84 (10)	81 (11)	.03
Age at menopause, years, mean (SD)	44.1 (5.1)	49.3 (5.8)	< .001
Diabetes, No (%)	13 (7.5)	12 (3.5)	.07
Smoker status, No (%)			
Never	60 (34.7)	132 (38.2)	.003
Former	95 (54.9)	95 (41.4)	
Current	18 (10.4)	70 (20.3)	
Antihypertensive medication, No (%)	60 (34.7)	1 (0.3)	< .001
Anticoagulant medication, No (%)	16 (9.2)	39 (11.4)	.54
Lipid-lowering medication, No (%)	31 (17.9)	85 (24.9)	.09
Education level, No (%)			.002
Primary	15 (8.7)	45 (13.0)	
Lower	67 (38.7)	172 (49.9)	
Intermediate	36 (20.8)	67 (19.4)	
Higher	15 (31.8)	61 (17.7)	
Total brain volume, mL, mean (SD)	902 (76)	900 (80)	.72
Age at cancer diagnosis, years, mean (SD)	42.6 (5.4)		
Time since diagnosis in years, mean (SD)	21.1 (4.4)		
Side of tumor, right side, No (%)	89 (51.4)		
Radiation field, No (%)			
Carotid artery not in radiation field^b^	17 (9.9)		
Carotid artery partly in radiation field^c^	102 (59.6)		
Carotid artery in radiation field^d^	52 (30.4)		

Side of tumor and radiation field were missing for one breast cancer survivor. SD = standard deviation

^a^In sitting position

^b^Axillary, breast, or chest wall radiation

^c^Internal mammary chain radiation

^d^McWhirter (supraclavicular and axillary) or supraclavicular radiation

We subsequently looked at the association of breast tumor side with plaque score by comparing carotid plaque scores of the left and right carotid artery within survivors. Here too, we used negative binomial regression models to investigate differences in carotid plaque scores. Linear regression models were used to determine the difference in IMT (total, left, and right), tCBF, and brain perfusion.

Statistical analyses were performed in R Version 3.3.2.

## Results

Sample characteristics are presented in Table 1. Breast cancer survivors had been diagnosed on average (SD) 21.1 (4.4) years before participation in this study, at a mean (SD) age of 42.6 (5.4) years. They had a higher systolic and diastolic blood pressure, a younger age at menopause (mean age 44.1 versus 49.3 years), a higher education level, and were less often current smokers than women from the reference group.

Breast cancer survivors had a similar total carotid plaque score (adjusted *β* = − 0.01 [95%CI − 0.20;0.18]) and total IMT score (adjusted *β* = 0.00 [95%CI − 0.02;0.03]) as cancer-free reference women (Table 2). Regarding brain perfusion, breast cancer survivors had a statistically significantly lower mean tCBF (adjusted *β* = − 19.6 ml/min [95%CI − 37.3;− 1.9]) and brain perfusion (adjusted *β* = − 2.5 ml/min per 100 ml [95%CI − 4.3;− 0.7]) than the cancer-free reference women (Table 2). Effect estimates for tCBF and brain perfusion hardly changed after including total mean IMT and total plaque score in the model (Table 2, Model III).

**Table 2:** Association between breast cancer survivors and carotid plaque scores, intima-media thickness, total cerebral blood flow, and brain perfusion

Outcome	Cancer-free reference women (n = 346)	Breast cancer survivors (n = 173)	Model I β (95% CI)^a^	Model II β (95% CI)^a^	Model III β (95% CI)^a^
Total carotid plaque score, median (IQR)	2.0 (1.0–3.0)	1.0 (0.0–3.0)	− 0.12 (− 0.31;0.07)	− 0.01 (− 0.20;0.18)	
Intima-media thickness, mean (SD)	0.83 (0.14)	0.84 (0.14)	0.00 (− 0.02;0.02)	0.00 (− 0.02;0.03)	
Total cerebral blood flow, ml/min, mean (SD)	547 (97)	520 (90)	− 19.5 (− 36.6;− 2.4)	− 19.6 (− 37.3;− 1.9)	− 19.7 (− 37.2;− 2.1)
Total brain perfusion, ml/min per 100 ml, mean (SD)	60.9 (9.5)	57.6 (9.0)	− 2.8 (− 4.6;− 1.1)	− 2.5 (− 4.3;− 0.7)	− 2.5 (− 4.2;− 0.7)

Model I = adjusted for age and body mass index; Model II = as Model I, plus: systolic blood pressure, diastolic blood pressure, prevalence of diabetes, smoking status, use of antihypertensive medication, anticoagulant medication, and lipid-lowering medication, and education; Model III = as Model II, plus: total mean intima-media thickness and total plaque score

*CI* confidence interval, *IMT* intima-media thickness, *IQR* interquartile range, *SD* standard deviation, *tCBF* total cerebral blood flow

^a^Difference in median plaque score, mean intima-media thickness, mean cerebral blood flow, or mean brain perfusion between cancer-free women (reference) and breast cancer survivors

Breast cancer survivors who underwent radiotherapy with a larger portion of the carotid artery in the radiation field had slightly higher carotid plaque scores, lower mean tCBF, and lower mean perfusion than those with a smaller portion of the carotid artery in the radiation field, albeit not statistically significant (Table 3). Also, they had a statistically significantly higher total and right IMT score (adjusted beta for total IMT score = 0.05 [95%CI 0.01;0.09] and for right IMT score = 0.09 [95%CI 0.01;0.16], Table 3). Right-sided breast cancer survivors had a statistically significantly lower left carotid plaque score (adjusted *β* = − 0.31 [95%CI − 0.60;− 0.02]) and a higher brain perfusion (adjusted *β* = 3.5 ml/min per 100 ml [95%CI 0.7;6.2]) than participants who survived a left-sided tumor (Table 4).

**Table 3:** Association between radiation to the carotid artery and carotid plaque scores, intima-media thickness, total cerebral blood flow, and brain perfusion in breast cancer survivors

Outcome	Small part carotid artery in radiation field (n = 102)	Carotid artery not in radiation field (n = 17)	Model I β (95% CI)^a^	Model II β (95% CI)^a^	Model III β (95% CI)^a^	Carotid artery in radiation field (n = 52)	Model I β (95% CI)^a^	Model II β (95% CI)^a^	Model III β (95% CI)^a^
Carotid plaque score, median (IQR)									
Total	1.0 (0.0–3.0)	2.0 (1.0–4.0)	0.35 (− 0.18;0.89)	0.22 (− 0.29;0.74)		1.5 (1.0–4.3)	0.31 (− 0.05;0.67)	0.27 (− 0.07;0.62)	
Left	1.0 (0.0–2.0)	1.0 (0.0–2.0)	0.15 (− 0.44;0.71)	0.05 (− 0.53; 0.60)		1.0 (0.0–2.0)	0.31 (− 0.07;0.68)	0.28 (− 0.09;0.64)	
Right	1.0 (0.0–2.0)	1.0 (0.0–3.0)	0.53 (− 0.04;1.09)	0.39 (− 0.15;0.91)		1.0 (0.0–2.0)	0.31 (− 0.09;1.09)	0.22 (− 0.17;0.61)	
Intima-media thickness, mean (SD)									
Total	0.82 (0.13)	0.84 (0.17)	0.01 (− 0.06;0.08)	0.00 (− 0.06;0.07)		0.89 (0.14)	0.06 (0.01;0.10)	0.05 (0.01;0.09)	
Left	0.83 (0.15)	0.84 (0.16)	0.03 (− 0.03;0.08)	0.03 (− 0.02;0.08)		0.87 (0.14)	0.05 (− 0.02;0.11)	0.05 (− 0.02;0.11)	
Right	0.81 (0.14)	0.85 (0.18)	0.03 (− 0.02;0.08)	0.02 (− 0.02;0.07)		0.90 (0.17)	0.10 (0.03;0.17)	0.09 (0.01;0.16)	
Cerebral blood flow, ml/min, mean (SD)									
Total	524 (91)	505 (62)	− 21.6 (− 66.7;23.5)	− 21.7 (− 67.5;24.2)	− 16.4 (− 61.7;28.8)	511 (92)	− 17.3 (− 47.2;12.5)	− 13.7 (− 44.9;17.5)	− 16.4 (− 61.7;28.8)
Brain perfusion, ml/min per 100 ml, mean (SD)									
Total	57.5 (9.1)	56.9 (6.6)	− 1.2 (− 5.8;3.5)	− 1.2 (− 5.9;3.5)	− 0.6 (− 5.2;4.0)	57.6 (9.3)	− 0.5 (− 3.6;2.5)	− 0.5 (− 3.7;2.7)	− 0.1 (− 3.3;3.1)

Model I = adjusted for age and body mass index; Model II = as Model I, plus: systolic blood pressure, diastolic blood pressure, age at menopause, prevalence of diabetes, smoking status, use of antihypertensive medication, anticoagulant medication, and lipid-lowering medication, and education; Model III = as Model II, plus: total mean intima-media thickness and total plaque score

*CI* confidence interval, *IMT* intima-media thickness, *IQR* interquartile range; *SD* standard deviation

^a^Difference in median total plaque score, mean intima-media thickness, mean total cerebral blood flow, or mean brain perfusion between breast cancer survivors treated with carotid artery partly in radiation field (i.e., internal mammary chain radiation, reference group), without carotid artery in radiation field, and with carotid artery in radiation field (i.e., McWhirter or supraclavicular lymph node radiation)

**Table 4:** Association between tumor location (left/right-sided breast cancer) and carotid artery plaque score in breast cancer survivors

Outcome	Left-sided breast cancer (n = 83)	Right-sided breast cancer (n = 89)	Model I β (95% CI)^a^	Model II β (95% CI)^a^
Carotid plaque score, median (IQR)				
Total	2.0 (1.0–3.5)	1.0 (0.0–3.0)	− 0.24 (− 0.56;0.09)	− 0.15 (− 0.36;0.05)
Left	1.0 (0.0–2.0)	1.0 (0.0–2.0)	− 0.34 (− 0.68;− 0.00)	− 0.31 (− 0.60;− 0.02)
Right	1.0 (0.0–2.0)	1.0 (0.0–2.0)	− 0.07 (− 0.43;0.29)	− 0.01 (− 0.36;0.35)
Intima-media thickness, mean (SD)				
Total	0.85 (0.13)	0.84 (0.15)	− 0.02 (− 0.05;0.02)	− 0.01 (− 0.05;0.03)
Left	0.85 (0.15)	0.84 (0.15)	− 0.02 (− 0.06;0.02)	− 0.01 (− 0.05;0.04)
Right	0.84 (0.14)	0.84 (0.17)	− 0.01 (− 0.06;0.03)	− 0.01 (− 0.06;0.03)
Cerebral blood flow, ml/min, mean (SD)				
Total	507 (84)	530 (92)	26.2 (0.29;52.1)	25.9 (− 0.90;52.7)
Brain perfusion, ml/min per 100 ml, mean (SD)				
Total	56.0 (8.1)	59.0 (9.4)	3.2 (0.6;5.9)	3.5 (0.7;6.2)

Model I = adjusted for age and body mass index; Model II = as Model I, plus: systolic blood pressure, diastolic blood pressure, age at menopause, prevalence of diabetes, smoking status, use of antihypertensive medication, anticoagulant medication, and lipid-lowering medication, and education

*CI* confidence interval, *IMT* intima-media thickness, *IQR* interquartile range, *SD* standard deviation

^a^Difference in median total plaque score, mean intima-media thickness, mean total cerebral blood flow, or mean brain perfusion between breast cancer survivors treated with left-sided cancer (reference) and right-sided cancer

## Discussion

This study shows that on average 20 years after treatment with chemotherapy and radiotherapy, breast cancer survivors have lower tCBF and brain perfusion than aged-matched cancer-free women. Our results within breast cancer survivors indicate that radiotherapy on a larger part of the carotid artery is associated with a greater IMT. Lastly, we found that plaque scores in the left carotid artery were significantly lower in participants with a right-sided tumor than in those with a left-sided tumor.

We found that breast cancer survivors had lower tCBF and brain perfusion than cancer-free women, which was not completely explained by carotid pathology. In contrast, it has previously been shown that 1 year after completion of chemotherapy, brain perfusion was increased in breast cancer survivors, which might reflect a temporary compensatory mechanism for chemotherapy-induced damage [31]. In addition, brain perfusion was decreased in the frontal and parietal parts of the brain, which was associated with lower gray matter density [32]. The lower brain perfusion in our study might therefore underlie the cognitive deficits and alterations in brain volumes that we previously observed in this group of cancer survivors [19, 20] and which are observed in breast cancer survivors who have completed chemotherapy in general [33]. We have explored this hypothesis in post-hoc analyses and indeed found that the relation between global cognitive function and brain perfusion differed between breast cancer survivors and reference women (Supplementary Material). In addition, lower brain perfusion is associated with a higher risk of transient ischemic attack (TIA) in the general population [34]. Although it has been shown that breast cancer survivors have a nonstatistically significant higher risk of TIA [35], it might be relevant to focus on those survivors with altered brain perfusion.

We did not find a difference in carotid pathology between the total group of breast cancer survivors and the cancer-free reference women. However, within breast cancer survivors, we found that more radiotherapy on the carotid artery was associated with a greater IMT. Carotid IMT is a marker for atherosclerosis. Although carotid plaques are a stronger predictor of cardiovascular disease than IMT in the general population [36], greater IMT is also associated with cardiovascular events independent of major cardiovascular risk factors including carotid plaques [37]. Therefore, greater IMT in breast cancer survivors treated with radiotherapy on the carotid artery might explain the higher risk of cardiovascular events in those breast cancer survivors who were treated with radiotherapy [1]. A potential explanation for the fact that we did not find differences between breast cancer survivors and cancer-free controls might be that cancer survivors had adopted a healthier lifestyle after their diagnosis and treatment. This may limit the damaging effects of chemotherapy and radiotherapy on the vascular system. This hypothesis is supported by a higher rate of former smokers in our group of cancer survivors, which might suggest that these women stopped smoking after their cancer diagnosis.

Our observation of higher left plaque score in breast cancer survivors with left-sided cancer than those with right-sided cancer may reflect an interaction between a generally higher rate of plaques in the left versus the right carotid artery and radiotherapy. In the general population, the prevalence of left-sided plaques is twice as high as right-sided plaques [38]. Also, left-sided plaques are predominantly composed of intraplaque hemorrhage and fibrous tissue and are therefore plaques more vulnerable to plaque rupture and subsequent thromboembolic complications than right-sided plaques [38]. In addition, the left carotid artery may be exposed to higher arterial pressure due to left–right differences in anatomy. For instance, the left carotid artery is directly connected to the aortic arch, whereas the right carotid artery is connected to the brachiocephalic artery [39]. It is therefore possible that radiotherapy accelerates the number of plaques on the left side. Previously, our group reported that breast cancer survivors who had received radiotherapy for left-sided breast cancer had higher risks for myocardial infarction (hazard ratio (HR) = 1.77) and congestive heart failure (HR = 1.41) than breast cancer survivors with right-sided tumors, although these effects were not significant [1]. This higher risk might be explained by a higher radiation exposure of the heart in left-sided cancer patients. Together, these findings emphasize the importance of cardiovascular risk screening in breast cancer survivors, in particular in those with left-sided breast cancer.

### Strengths and limitations

Strengths of our study are the sample of almost two hundred breast cancer survivors with a long interval since radiotherapy and chemotherapy, the homogeneous study population with regard to the cytotoxic agents received (regimen, cycles), and the comparison with population-based reference women without a history of cancer who underwent the same examinations as the breast cancer survivors.

CMF chemotherapy has a high likelihood of inducing early menopause. Age at menopause was therefore considered a mediating variable in our between-group analyses. Because of this, it is impossible to separate the direct effects of chemotherapy and the effects through menopause. Samples with sufficient numbers of subjects who did and did not reach early menopause due to chemotherapy are necessary to separate the effects of chemotherapy and menopause on vascular pathology and brain perfusion.

A limitation is that the included breast cancer survivors did not receive endocrine therapy. Endocrine therapy was not part of the standard treatment for patients with breast cancer in the Netherlands until the mid-1990s. However, nowadays, patients frequently receive endocrine therapy, and it has been shown that this therapy is associated with the presence of carotid plaques [40]. Also, the CMF regimen is no longer considered an optimal adjuvant chemotherapy regimen for breast cancer, but it has been the standard regimen worldwide up to the 1990s [39]. Therefore, it is currently the only regimen that enables the investigation of the very late effects of chemotherapy in sufficiently large numbers of persons. Current regimens often include individual components of the CMF regimen, including cyclophosphamide and 5-fluorouracil. Therefore, the current findings may also be relevant for breast cancer survivors that are treated with contemporary chemotherapy regimens. Also, there is still a large group of women who have been treated with CMF in the past of whom some women may now experience the negative cerebral consequences. Lastly, the observed associations may be less pronounced in breast cancer patients who are currently treated with radiotherapy, as radiotherapy for breast cancer is usually given to more limited target volumes and radiotherapy techniques have improved leading to lower doses to the carotid arteries [41] and the heart [42].

## Conclusion

Breast cancer survivors have lower brain perfusion on average 20 years post-treatment which may be part of the mechanism underlying the well-known cognitive sequelae of chemotherapy. Radiotherapy on the carotid artery is associated with a larger IMT, which in turn might result in more cardiovascular disease. Therefore, cardiovascular risk management of breast cancer survivors is important.
